# Supplementary figures and images for: Epac activation reduces trans-endothelial migration of undifferentiated neuroblastoma cells and cellular differentiation with a CDK inhibitor further enhances Epac effect
Source: PLoS One. 2024 Nov 4;19(11):e0304547. doi: 10.1371/journal.pone.0304547 (PMC11534210; doi:10.1371/journal.pone.0304547)

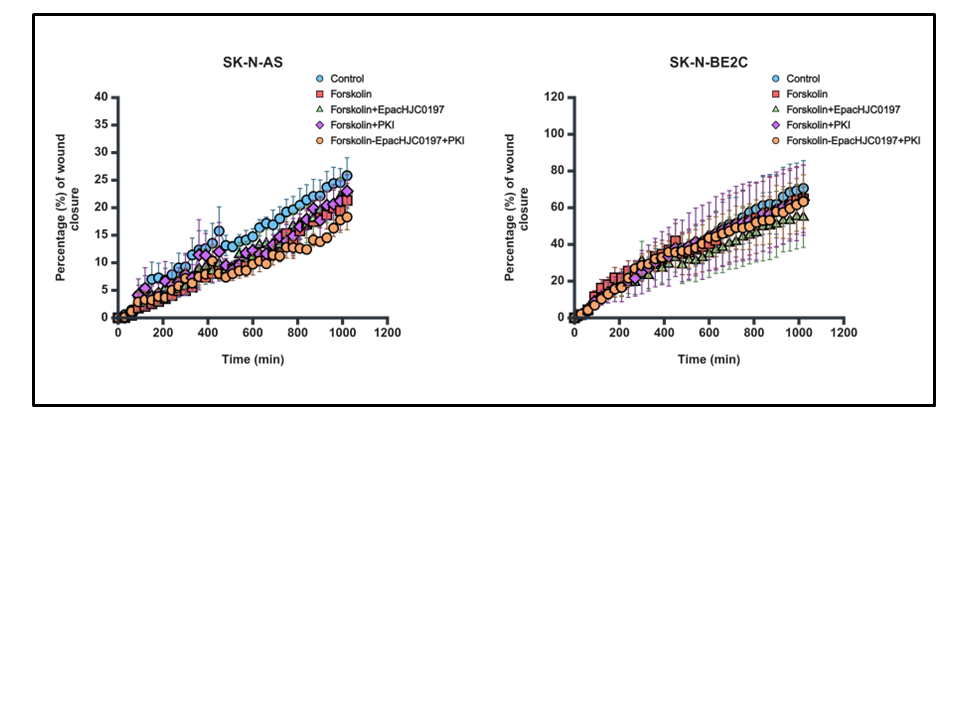

Supplement: S1 Fig — Comparison of SK-N-BE2C and SK-N-AS cells in wound closure assay. The cells were treated with either forskolin (10 μM) alone or with forskolin (10 μM) and Epac antagonist (HJC0197 (5 μM) or PKI (5 μM)). No difference was observed between the forskolin-treated and untreated SK-N-BE2C and SK-N-AS cells. Wound treated with PKA inhibitor and Epac antagonists did not change the speed of migration. Each plot in graphs for SK-N-BE2C and SK-N-AS represents the mean ±SEM. This experiment was repeated three times (N = 3). (TIF) [file pone.0304547.s001.tif]

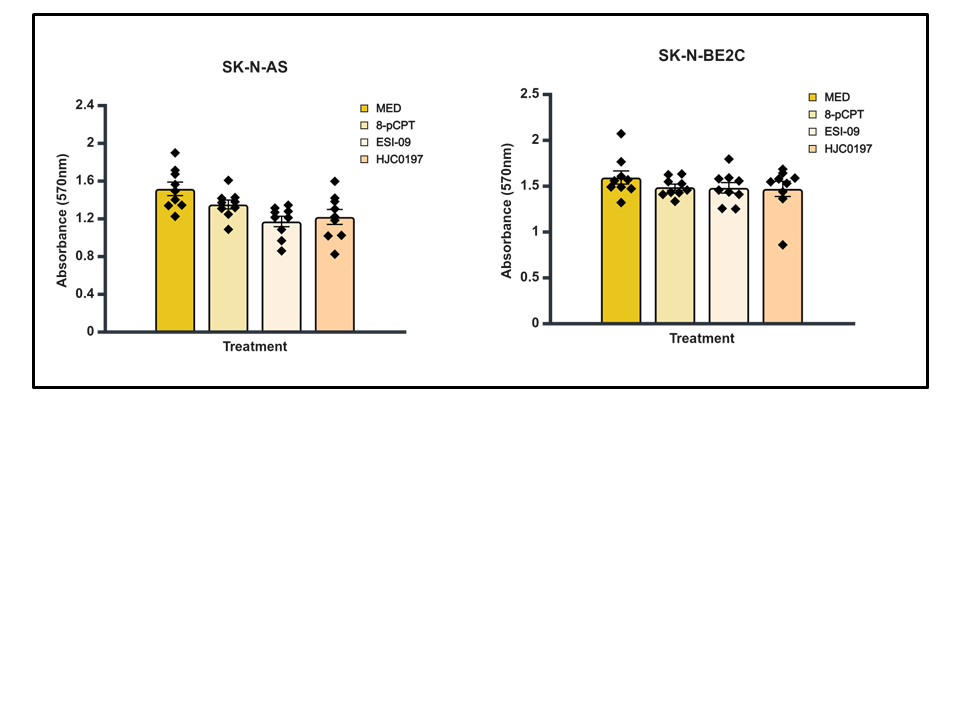

Supplement: S2 Fig — SK-N-BE2C and SK-N-AS cells were seeded into a flat bottom 96 well plate for 24 hours. Cells were then treated with 8-pCPT (10 μM), ESI-09 (5 μM) and HJC0197 (5 μM) for 24 hours and MTT absorbance were measured. 8-pCPT, as well as ESI-09 and HJC0197 treatment, did not cause any clear change in the cell number in both cell lines compared to the control (MED). Each bar represents the mean ± SEM. One way ANOVA was used to compare the effect of treatment on NB cells proliferation. Means comparison was assessed by Tukey post-test. P > 0.05. This experiment was repeated three times (N = 3). (TIF) [file pone.0304547.s002.tif]
