# Supplementary material for: Epac activation reduces trans-endothelial migration of undifferentiated neuroblastoma cells and cellular differentiation with a CDK inhibitor further enhances Epac effect
Source: PLoS One. 2024 Nov 4;19(11):e0304547. doi: 10.1371/journal.pone.0304547 (PMC11534210; doi:10.1371/journal.pone.0304547)
Supplement: S1 Appendix — (DOCX) [file pone.0304547.s003.docx]

**Appendix**

**Scratch migration assay**

For the scratch assay, 5 x 10^5^ cells of both SK-N-BE2C and SK-N-AS cells in 500 µl of culture medium were plated per well using a 24 well culture plate. Cells were cultured to form a 100% confluent monolayer within 24 hours. For the initial part of the experiment, the wound was generated on the monolayer cells using a micropipette tip. Then the culture medium was removed from the well and the generated wound rinsed with 600 µl of fresh culture medium. Fresh 500 µl of culture medium was then added to the rinsed monolayer of cells. Subsequently, pictures were captured manually using Leica dissecting microscope at 0, 2, 4, 6, 8 and 24 hours. In later experiments, the wound was generated on 100% confluent monolayer cells in a 24 well plate and the plate mounted on Cell IQ machine for imaging set at 30-minute intervals over 24 hours. Four positions were selected and added per well using the Cell IQ software before allowing it to run. Pictures were analysed with Image J for the manual experiments, and Cell IQ image analysis software was used to analyse all experiments run on the Cell IQ machine. All experiments were done in triplicates.

**MTT proliferation assay**

SK-N-BE2C or SK-N-AS cells were cultured, and a preliminary experiment was set to determine the appropriate cell density for the MTT assay. Following optimisation, 1.25 x 10^6^ cells per ml solution of each cell line were prepared. Using a flat bottom 96 well plate, 100 µl of prepared SK-N-BE2C cells or SK-N-AS cells were plated per well in triplicate. 3 blank control wells were prepared with 100µl of the cell culture medium only. The prepared cultured plate/s were then incubated overnight for24 hours. At the end of incubation 10 µl of 5 mg/ml MTT reagent was added to all the wells in the dark. Each of the plates was then wrapped with aluminium foil and incubated for 4 hours at 37^o^C and 5% CO_2._ A purple precipitate became visible at the end of 4 hours incubation, then 100µl of 10% 0.01M SDS was added to all the wells, control inclusive, in the dark environment, at the same time. The plate was then covered again with aluminium foil and incubated at 37^o^C and 5% CO_2_ overnight. Absorbance was read at 570 nm wavelength using Floustar Omega plate reader. Values from triplicate experiments were averaged for each reading and analysis performed subsequently.

#### An Epac antagonist HJC0197 and PKI do not alter SK-N-BE2C and SK-N-AS response to forskolin in scratch wound assays.

To investigate the effect of forskolin and Epac and PKA agonist on NB cell migration we employ the use of a pharmacological agents in form of forskolin and 5-Cyano-6-oxo-1,6-dihydroyclopentyl (HJC0197). HJC0197 is an Epac antagonist with a cyclic nucleotide-binding domain (CNBD) binding site of Epac1/Epac2 [20,21]. Alternatively, 3-(5-Tert-butyl-isoxazol-3-yl)-2-[(3-chlorophenyl)-hydraznol]-3-oxopropionitrile (ESI-09) which is also another an Epac antagonist with CNBD-B binding site could be employed [22,23]. The two aforementioned agents inhibit the activity of Epac independently without interfering with the activity of that of PKA sensors. HJC0197 was used at a final concentration of 5 µM which falls within the range of published IC_50_ values (5.9µM) [9]. PKI, a protein kinase A-inhibitor in myristoylated amide form, was used at a concentration of 5 µM.

The scratch wound was treated with either forskolin only or with the combination of either forskolin and Epac antagonist, or forskolin and myristoylated PKI, and the rate of wound closure was assessed within 24 hours (**S1 Fig**). No significant difference was observed in the rate of wound closure between the forskolin-treated cells with or without either PKA inhibitor or HJC0197 in both cell lines during the course of treatment.

**Effect of 8-pCPT, ESI-09 and HJC0197 on the proliferation of NB**

To investigate the effect of activation of Epac on the proliferation of NB cells. Cells were treated using an Epac agonist (8-pCPT (10 µM)) and antagonists (ESI-09 and HJC0197). 10 µM of 8-pCPT was applied for 24 hours to SK-N-BE2C and SK-N-AS cells. In parallel, the Epac antagonists ESI-09 and HJC0197 were also applied individually to SK-N-BE2C and SK-N-AS cells for 24 hours each at a final concentration of 5 µM. Untreated cells were used as control (MED). MTT absorbance was measured using plate reader at the end of 24 hours treatment for all the groups. Treatment with either 8-pCPT, ESI-09 or HJC0197 did not cause any observable difference on the MTT absorbance of both SK-N-BE2C and SK-N-AS relative to the control in both cell lines (**S2 Fig**). Data analysis by means of one way ANOVA shows that there was no significant statistical difference in terms of proliferation between the treatment and control for both SK-N-BE2C and SK-N-AS. P > 0.05.

**Effect of forskolin on the invasion of SK-N-BE2C and SK-N-AS cells**

For the invasion study, we employ the use matrigel invasion assay. Tumour spheroids generated for SK-N-BE2C and SK-N-AS cells and embedded in Matrigel were treated with 10 µM forskolin for 48 hours with DMEM used as a control. Images of Matrigel embedded tumour spheroids were captured at 0, 24 and 48 hours respectively. Image J software was then subsequently used to draw an outline of the area of tumour spheroids for the control and treatment. Analysis of the drawn area does not show any significant difference between the treated spheroid and the control. Suggesting that forskolin may not have a demonstrable effect on the process of Matrigel invasion of SK-N-BE2C and SK-N-AS cells.
